# Complete Generalization of the Equations for the Stress–Strain Curves of Concrete under Uniaxial Compression

**DOI:** 10.3390/ma16093387

**Published:** 2023-04-26

**Authors:** Vanesa Domínguez-Cartes, Daniel Ramos-Cabeza, María Luisa de la Torre, Francisco Salguero-Andújar

**Affiliations:** 1 Department of Mining, Mechanical, Energy and Construction Engineering, Campus de El Carmen, School of Engineering, University of Huelva, 21007 Huelva, Spain; vanesa.dominguez@dimme.uhu.es (V.D.-C.); daniel.ramos@dimme.uhu.es (D.R.-C.); mltorre@dimme.uhu.es (M.L.d.l.T.); 2 Centro Científico Tecnológico de Huelva (CCTH): Science and Technology Research Center of Huelva (STRC-Huelva), Faculty of Experimental Sciences, Campus de El Carmen, University of Huelva, 21007 Huelva, Spain

**Keywords:** concrete, modeling, elastic moduli, mechanical properties, characterization

## Abstract

The existence of more than thirty stress–strain equations, including those proposed by the government regulations in many countries, seems to indicate that additional, unifying, and at the same time generalizing research is necessary for this subject. Many expressions can be found to set or determine the initial modulus of elasticity of concrete, i.e., the modulus of elasticity of concrete when no load has been applied to it. This work proposes a complete generalization of the equations based on scalar damage models, applicable to all types of concrete tested under uniaxial compression with any constant rate of stress or strain, although in no case can it be considered a constitutive model. We prefer to discuss an equation that models the shape of the stress–strain curve. Thus, the shape of this curve is studied here in the same way a forensic scientist would, which is why we could see this work as an autopsy carried out on the test specimen through the trace left in the plane *σ*-*ε* by the straining process up until its inevitable outcome. That is to say, we believe in a purely phenomenological approach. The results are compared with the data obtained experimentally by analyzing test specimens made using various mixed portions of cement, water, and aggregates.

## 1. Introduction

The search for a mathematical expression to represent the stress–strain evolution that a concrete test specimen submitted to uniaxial compression stress undergoes is almost as old as the concept of reinforced concrete as a building material itself. Since the pioneering proposals of Bach [1] in 1897 and Ritter [2] in 1899 veritable dynasties of equations based on three significant main approaches, which we will review briefly below, have been built up.

On the one hand, we find proposals centered around the 1956 equation by Smith and Young [3], which was written as follows:

(1) $$\sigma =\frac{f_{c}^{\prime }}{\varepsilon_{0}}\exp\left[1-\frac{\varepsilon }{\varepsilon_{0}}\right]$$

where *σ* is the applied stress, $f_{c}^{\prime }$ is the maximum stress the test specimen reaches, $\varepsilon_{0}$ is the strain this test specimen undergoes at the point of maximum stress, and ε is the strain.

If we follow Yip [4] and develop Equation (1) as a power series, we obtain the following:

(2) $$\sigma =\frac{f_{c}^{\prime }\frac{\varepsilon }{\varepsilon_{0}}\exp\left[1\right]}{\left[1+\left(\frac{\varepsilon }{\varepsilon_{0}}\right)+\frac{1}{2}{\left(\frac{\varepsilon }{\varepsilon_{0}}\right)}^{2}+\frac{1}{6}{\left(\frac{\varepsilon }{\varepsilon_{0}}\right)}^{3}+\cdots \right]}$$

which is why, according to the findings of Yip, the proposals by Desayi and Krishnan [5], Saenz [6] in 1964, and Alexander [7] in 1965 are the result of the truncation of a varying number of terms of the denominator in Equation (2). In the same way, the proposals by Tulin and Gerstle [8], Popovics [9] and Carreira and Chu [10] represent a generalization of the aforementioned proposal by Desayi and Krishnan, which consists of truncating all the terms in the denominator in Equation (2) except $1+{\left(\varepsilon /\varepsilon_{0}\right)}^{2}$ and substituting the quadratic exponent with another, *n*, which is more general and would vary according to the characteristics of the concrete being tested. A subsequent generalization, to use his own words, of this type of equation is represented by Tsai’s 1988 proposal [11], while, for their part, Collins, Mitchell, and MacGregor, [12] or Tasnimi [13] use similar expressions to those of Popovics or Saenz, but they modify the exponent depending on whether the function models the ascending or descending branch of the stress–strain curve.

Elsewhere, the proposal formulated in 1971 in Sargin’s doctoral thesis [14] corresponds to equations of the following type:

(3) $$Y=\frac{AX+BX^{2}}{1+CX+DX^{2}}$$

where $Y=\sigma /f_{c}^{\prime }$, $X=\varepsilon /\varepsilon_{0}$, and *A*, *B*, *C*, and *D* are material constants. Another series of equations appears to be based on rational functions, among which the 1978 reinterpretation by Wang, Shah, and Naaman [15] stands out, in which two equations, or rather, two variants of the same equation are proposed, each different depending on whether they model the ascending or descending portion of the curve. Indeed, the authors state that the accuracy of Equation (3) improves greatly when *A*, *B*, *C*, and *D* take different values according to whether they evaluate the ascending or descending branch of the curve, instead of varying the denominator exponent. The various options of the Model Code [16], which in 1990 proposed a variant of Sargin’s general equation, are ascribed to this line, specifically, the 1969 proposal by Sargin and Handa [17] for the ascending branch of the curve and part of the descending branch, although this is declared valid only for concretes with a characteristic strength that is under 80 MPa. The 1990 Model Code was partially reformed in 1995 by CEB Bulletin no. 228 [18], in which (following the model proposed by Gysel and Taerwe [19]), the validity interval of the equations was increased to 50 MPa ≤ $f_{c}^{\prime }$ ≤ 100 MPa. The same equation as the 1990 Model Code is proposed for the ascending branch (now it is only valid up to the peak stress $f_{c}^{\prime }$). The equation for the descending branch is modified by means of the following expression:

(4) $$\sigma =\frac{f_{c}^{\prime }}{1+{\left(\frac{\varepsilon -\varepsilon_{0}}{\eta -1}\right)}^{2}}$$

where $\eta =\varepsilon_{0}+t$, and the parameter *t* is determined experimentally for each characteristic strength of the concrete. The 2010 Model Code returns to the equations in the 1990 code, with the difference that their applicability is now widened to concretes with a characteristic strength of 120 MPa and for the first time includes the damage model as a valid approach to describe the non-linear triaxial behavior of concrete.

Finally, we will refer to the equations based on damage models. Originally proposed by Kachanov [20] in 1956, subsequently modified by Rabotnov [21], and mainly developed from the 1980s onwards [22,23,24,25], Continuous Damage Mechanics (CDM) has been widely accepted to simulate the complex fundamental behavior of many materials used in engineering. In particular, the models based on an internal damage variable represented by a scalar function are characterized by their simplicity of implementation and versatility but do not imply that the damage is isotropic since they can follow the weakest link failure criterion. This damage variable reflects the material’s level of deterioration as it is stretched and transforms the real stresses into effective stresses, in such a way that a general equation that relates stresses to strains can be written in the following form:

(5) $$\sigma =\Psi \left(\varepsilon \right)\left[1-\omega \left(\varepsilon \right)\right]$$

where $\Psi \left(\varepsilon \right)$ represents the response of the undamaged material and $\omega \left(\varepsilon \right)$ a scalar damage function which varies between 0 (when the material has not been stressed yet) and 1 (when the collapse of this material occurs).

The proposals we mention below assume that the function $\omega \left(\varepsilon \right)$ corresponds to a cumulative distribution function (CDF) for the probability of the damage occurring in the particles of the material and differ from each other in terms of the type of statistical distribution chosen to simulate the evolution of this damage according to the strain imposed by the test. Specifically, the equation proposed by Shah and Winter [26] in 1966 is written as follows:

(6) $$\sigma =K_{1}\varepsilon \exp\left[-{\left(\frac{K_{1}\varepsilon -2}{K_{2}}\right)}^{m}\right]$$

where *K*_1_, *K*_2_, and *m* are to be determined experimentally. From our point of view, this article is of paramount importance, since the exponential formula (also used by Smith and Young with slight differences and *m* = 1) is deduced in it for the first time, by means of Weibull’s well-known statistical theory of the strength of materials [27,28]. Weibull’s theory is potentially linked to the concepts proposed by Griffit [29] and Smekal [30]. Taylor [31,32,33] suggests that weak zones in a material can contribute to its failure, whether they are microcracks or dislocations in the atomic mesh. In the early 1930s, Orowan, Poliani, and Taylor separately concluded that the plastic strain of ductile materials could be explained by the theory of dislocations developed by Volterra in 1921. If we assume that these weak zones will break as soon as they reach a volume subjected to *σ* stress and that there are *n* weak zones per volume unit, and the *σ* stress is concentrated within a small volume *dv*, then the probability of breakage for the volume element can be expressed as *dP* = *ndv*. If there are *N* elements of volume *dv*, and the probability of breakage is *P*, then the probability of survival can be calculated as *S* = 1 − *P* = (1 − *dP*)*^N^* = (1 − *ndv*)*^N^*, where resistance would decrease due to the presence of numerous weak zones in the material, so

(7) $$P=1-{\left(1-ndv\right)}^{N}$$

The total volume subjected to stress wil be *V* = *Ndv* and

(8) $$P=1-{\left(1-\frac{nV}{N}\right)}^{N}=1-{\left(1-\frac{nV}{N}\right)}^{\left(N/nV\right)nV}$$

However, if *N* increases as *dv* decreases indefinitely until *V* becomes constant, the following is obtained:

(9) $$P=1-\underset{\frac{N}{\mathrm{nV}}\to \infty }{\lim}{\left(1-\frac{\mathrm{nV}}{N}\right)}^{\left(N/\mathrm{nV}\right)\mathrm{nV}}=1-e^{-nV}$$

In the latter part of his influential article, he asserts that Equation (9) is purely theoretical and lacks physical significance unless a function of the material, ∫*ω*(*ε*)*dv* = *nv*, can be experimentally determined. Such an expression may be deemed valid with a certain degree of confidence for a particular material, as the results of experiments may reveal the existence of a characteristic distribution function for each unique material. By utilizing such experimental data, it becomes possible to derive the mathematical expression for the function.

To this end, he selected a coordinate system that he deemed most suitable for computing the distribution function corresponding to Equation (9), where the probability of fracture was $P=1-e^{-\int \omega \left(\varepsilon \right)dv}$. At this juncture, he introduced his most renowned proposition: the hefunction *ω*(*ε*) should be chosen as

(10) $$\omega \left(\varepsilon \right)={\left(\frac{\varepsilon -\varepsilon_{a}}{\varepsilon_{0}}\right)}^{m}$$

As Weibull heralded, this equation allows the data to be the ones that provide the parameters of the distribution function.

On this basis, concrete could be thought of as a material composed of a very large number of tiny structural units. The individual strength of each element could be determined by a breaking test, although if this test were repeated for each element, the value of the breaking load would not always be exactly the same, but would present some dispersal around a central mean value. Therefore, it would not be possible to indicate a precise value for the breaking load for each element, although it would be possible to indicate a definite breaking probability for each value *σ* of the applied stress. Shah and Winter’s idea was to adopt the probability distribution function proposed by Weibull in 1939 for the strength of elemental structural units, which concrete would be composed of, so that as the latter is loaded, the weakest units would break first, while those with the greatest strength would continue resisting, resulting in a redistribution of stresses and a process of progressive breaking.

For their part, the series of three articles [34,35,36] that Blechman published between 1988 and 1989, stand out, more than for the equations proposed, for their theoretical considerations of the process of concrete breaking in the uniaxial compression test. Specifically, first of all, Blechman proceeds from the firm conviction that the attempt to find a simple, single equation to model the complete stress–strain curve of concrete is a pointless exercise, due to the fact that three essentially different states are assembled in it, namely, an initial perfectly elastic phase up to a certain strain $\varepsilon_{a}$, another in which a growing phenomenon of *atrophy* of the strength characteristics of the material is produced up to the strain at the peak stress, $\varepsilon_{0}$, and, lastly, the final phase where a process of macroscopic destruction occurs, in which the concrete no longer accepts any increase in load. For this reason, he divides the stress–strain curve into three sections. The first, governed by Hooke’s law up to a certain strain value, the second would represent the aforementioned atrophy process of the strength characteristics of concrete and which we can identify with an isotropic damage model, whose distribution function would now be Lord Rayleigh’s probability density function, and the third, modeled by a complex incremental algorithm. Blechman takes as his starting point the basis that, while in the section between the elastic limit strain $\varepsilon_{a}$, and the maximum limit strain $\varepsilon_{0}$, the microcracks in the stressed granules do not affect their macro-continuity or macro-rigidity, in the descending branch, the situation changes drastically, resulting in the appearance and growth of macroscopic cracks (observable to the naked eye), reducing the overall rigidity of the piece and, in short, producing a growing destruction mechanism, governed fundamentally by the means of applying stress or strain using the test machine. For this reason, he considers the concept of stress inapplicable in this phase, replacing the stress–strain relationship with the strain–response relationship of concrete, ruled by two *macro-functions*. These two macro-functions could only be obtained, according to Blechman, by means of the iterative numerical algorithm that he presents in the third article in the series.

Finally, it is worth noting the contributions of Ferretti et al. [37,38] in which an experimental instead of a numerical approach is followed to identify the unified stress–strain curve in uniaxial compression in which the failure rate (a concept that we will address later in this work), both under load conditions of constant stress increase as of constant strain rate increase, can increase, be constant, or decrease with strain.

## 2. Materials and Methods

### 2.1. Generalized Extreme Value Distribution

In statistics, Extreme Value Theory analyses the conditions under which the extremes of a random sample converge towards a non-degenerate limit distribution when the size of the sample, n, tends towards infinite numbers of variables. The development of the fundamental models of this theory is due to Fréchet [39], Fisher and Tippett [40], and Gnedenko [41], among others. The Fisher–Tippett–Gnedenko theorem (also known as the Fisher–Tippet theorem or the Extreme Value Theorem) is a general result of the Extreme Value Theory, which led to the development of asymptotic distribution to model maxima (or minima), called Generalized Extreme Value Distribution (GEVD). In Extreme Value Theory, this theorem plays the same role as the Central Limit Theorem in the study of means. It can be formulated as follows:

Let *X*_1,*n*_ ≥ … ≥ *X*_i,*n*_ ≥ … ≥ *X_n,n_* be a set of identically distributed, independent, random variables with a common distribution function *F*, i.e., *F*(*x*) = *P*(*X_i,n_* ≤ *x*), and let *M_n_* be the maximum of all of them, i.e., *M_n_* = *X*_1,*n*_ = max(*X*_1,*n*_ ≥ … ≥ *X_i,n_* ≥ … ≥ *X_n,n_*), which will have the following distribution function:

$$P\left(M_{n}\leq x\right)=P\left(X_{1,n}\leq x;\cdots ;X_{i,n}\leq x;\cdots ;X_{n,n}\leq x\right)=\prod_{i=1}^{n}P\left(X_{i,n}\leq x\right)=F\left(x\right)$$

The Stability Postulate establishes that to obtain a non-degenerate limit distribution, it is necessary to reduce the maximum by applying a linear transformation with coefficients $a_{n}\in R,$ *b_n_* > 0 which depend only on the size of the sample *n*, i.e.,

(11) $$P\left(\frac{M_{n}-a_{n}}{b_{n}}\leq x\right)=F^{n}\left(a_{n}+b_{n}x\right)=F\left(x\right)$$

and it is said that *F*(*x*) is a *max*-*stable* distribution.

So, if the constants $a_{n}\in R,$ *b_n_* > 0 exist, this means that

(12) $$G\left(x\right)=\underset{n\to \infty }{\lim}P\left(\frac{M_{n}-a_{n}}{b_{n}}\leq x\right)$$

where *G*(*x*) is a non-degenerate asymptotic distribution which represents the GEVD function and which belongs to some of the following families: Gumbel distribution [42], Fréchet distribution, or Weibull distribution.

More accurately, it could be said that Gumbel, Fréchet, and Weibull distributions are particular cases of a general asymptotic distribution called Generalized Extreme Value Distribution, the CDF of which takes the following form:

(13) $$G(x;a\_n,b\_n,\xi )=1-\exp\left[-{\left(1+\frac{1}{\xi }\left(\frac{x-a_{n}}{b_{n}}\right)\right)}^{-\xi }\right]$$

Indeed, if we take *ξ* = 0 in Equation (13), the GEVD is not defined, but the limit is as follows:

$$\underset{\xi \to 0}{\lim}{\left[1+\frac{1}{\xi }\left(\frac{x-a_{n}}{b_{n}}\right)\right]}^{-\xi }=\exp\left[-\left(\frac{x-a_{n}}{b_{n}}\right)\right]$$

Then, the following is obtained:

(14) $$GEVD_{I}=1-\exp\left[-\exp\left[-\left(\frac{x-a_{n}}{b_{n}}\right)\right]\right]$$

which is a type I GEVD or Gumbel distribution.

Elsewhere, taking *m* = *ξ*, we obtain the following:

(15) $$GEDV_{II}=1-\exp\left[-{\left(\frac{x-x_{a}}{x_{0}}\right)}^{-m}\right]$$

which is Fréchet distribution with *x_a_* = *a_n_* + *mb_n_* and *x*_0_ = *mb_n_*, which fulfill the Stability Postulate (Equation (13)).

Lastly, this results in *m* = −*ξ*:

(16) $$GEDV_{II}=1-\exp\left[-{\left(\frac{x-x_{a}}{x_{0}}\right)}^{m}\right]$$

which is the more usual expression of the Weibull distribution or type III GEVD.

### 2.2. Fractional Calculus

#### 2.2.1. Introduction

Fractional calculus is a field of mathematical studies, which until now has been relatively far-removed from the Strength of Materials, and which arises from the classical definitions of differential and integral calculus operators in a similar way to how fractional exponents are considered an extension of whole-number exponents.

Specifically, we will consider the meaning of the exponent as our primary school teachers tell us to consider it. Exponents provide an abbreviated notation for what is a repeated multiplication of a numeric value by itself. However, this *physical* definition becomes confusing if we consider non-integer exponents before logarithms are introduced in secondary school. While anyone can understand that *x*^5^ = *x*·*x*·*x*·*x*·*x*, i.e., the quantity *x* multiplied by itself five times, how could we describe the actual meaning of *x*^2.3^? Or, even worse, what is the physical meaning of the transcendental exponent *x*^π^? It is not easy to imagine what it would be to multiply something or a quantity by itself 2.3 times, or π times and yet these expressions have a definite value for any value of *x* which is verifiable by means of infinite developments in series or, as is more common nowadays, using a calculator.

**Figure 1:**
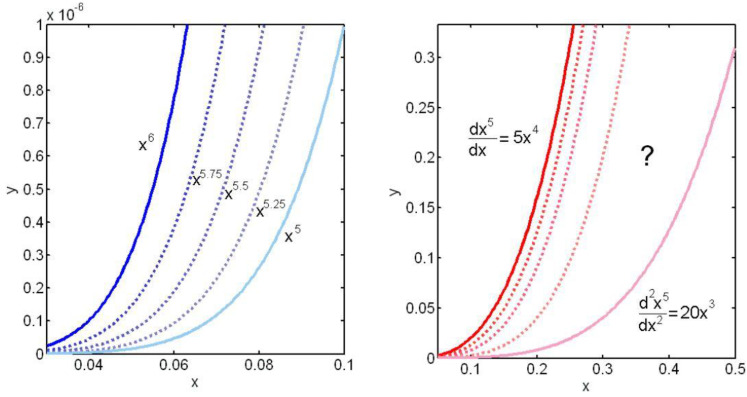
Analogy between exponents and fractional-order derivation. (The blue toned lines represent power functions, while the red toned lines represent fractional order derivatives? Is it possible to completely fill the plane with derivatives of fractional order in the same way that we do it with power functions?).

To illustrate our reasoning, we will use Figure 1. On the left, we represent the graph of the functions *f*_1_(*x*) = *x*^5^ and *f*_2_(*x*) = *x*^6^. The fractional exponents appear as functions that occupy the *intermediate* positions between *f*_1_ and *f*_2_. On the right side are represented the first and second derivatives of *f*_1_(*x*) = *x*^5^, i.e., *f*′_1_(*x*) = d*x*^5^/d*x* = 5*x*^4^ and *f*″_1_(*x*) = d^2^*x*^5^/d*x*^2^ = (d/d*x*) (d*x*^5^/d*x*) = d5*x*^4^/d*x* = 20*x*^3^.

Now, we could ask the following question: would it be possible to define *other fractional-order derivatives* between 1 and 2 that occupy the intermediate positions between d*x*^5^/d*x* and d^2^*x*^5^/d*x*^2^ in a similar way to how the fractional exponents between 5 and 6 are defined?

In the matter of the birthplace of the concept of fractional calculus, the majority of authors say that it was first formulated in 1695 by Guillaume de l’Hôpital in a letter to Gottfried Wilhelm Leibniz in which he showed an interest in the meaning of Leibniz’ notation d*^n^y*/d*x^n^* (the most popular nowadays) for the derivative of order $n\in N_{0}≔\left\{0,1,2,\cdots \right\}$. What would happen if *n* = 1/2? (asked l’Hôpital). In his reply, dated 30 September 1695, Leibniz answered l’Hôpital with the following: *…This is an apparent paradox from which, one day, useful consequences will be drawn*.

In addition to the theory of differential, integral, and integrodifferential equations, and the special functions of mathematical physics, as well as their extensions and generalizations in one or more variables, some of the areas of the current application of fractional calculus include the flow of fluids, rheology, the dynamic processes of porous structures and self-similar objects (fractals), fuzzy transport theory, electrical networks, probability and statistics, dynamic system control theory, viscoelasticity, corrosion electrochemistry, physical chemistry, optics, signal-processing, etc.

#### 2.2.2. Definition of Fractional-Order Integral

We take Cauchy’s formula as a starting point to calculate the iterated integral:

$$D^{-n}f\left(x\right)=\iint \cdots \int f\left(x\right)dx=\frac{1}{\left(n-1\right)!}\int_{0}^{x}f\left(t\right){\left(x-t\right)}^{n}dt$$

The following constitutes a generalization of the notion of *n*! introduced by Euler by means of the gamma function:

$$\Gamma \left(z\right)=\int_{0}^{\infty }t^{z-1}\exp\left[-t\right]dt\Rightarrow \Gamma \left(z\right)=\left(z-1\right)!$$

where *z* is any complex number with Re(*z*) > 0. Liouville [43] arrived at the integral which bears his name and generalized the natural number *n* to any number *α* with Re(α) > 0, which gives rise to the first formal expression of a (non-integer) fractional-order integral:

(17) $$D^{-\alpha }f\left(x\right)=\frac{1}{\Gamma \left(\alpha \right)}\int_{0}^{\infty }\frac{f\left(t\right)dt}{{\left(x-t\right)}^{1-\alpha }}$$

Later, Riemann, as a student, modified or generalized the Liouville integral, changing the lower limit 0 to *a* and the upper limit *x* to *b*, giving way to the c integrals, which constitute the definition we will use for fractional integration:

(18) aDx−αfx=1Γα∫axftdt(x−t)1−α;(x>α;Re(α)>0)

(19) xDb−αfx=1Γα∫xbftdt(x−t)1−α;(x>b;Re(α)>0)

where *_a_D_x_*^−*α*^*f*(*x*) and *_x_D_b_*^−*α*^*f*(*x*) are called Riemann–Liouville fractional integrals on the left and the right, respectively.

#### 2.2.3. Fractional Calculus and Constitutive Models

We could say that the simplest constitutive models are Hooke’s law

(20) $$\sigma \left(t\right)=E\varepsilon (t)$$

for perfectly and indefinitely elastic solids and the equation of Newtonian ideal fluids:

(21) $$\sigma \left(t\right)=\eta \frac{d\varepsilon (t)}{dt}$$

where *E* and *η* are constants.

Equations (20) and (21) represent mathematical models for solid materials and ideal fluids, respectively, and neither of them exists in nature. In fact, real materials combine the behaviors of these two limited cases, remaining somewhere in the middle between the ideal solids and fluids, if we order the materials regarding their consistency.

**Figure 2:**
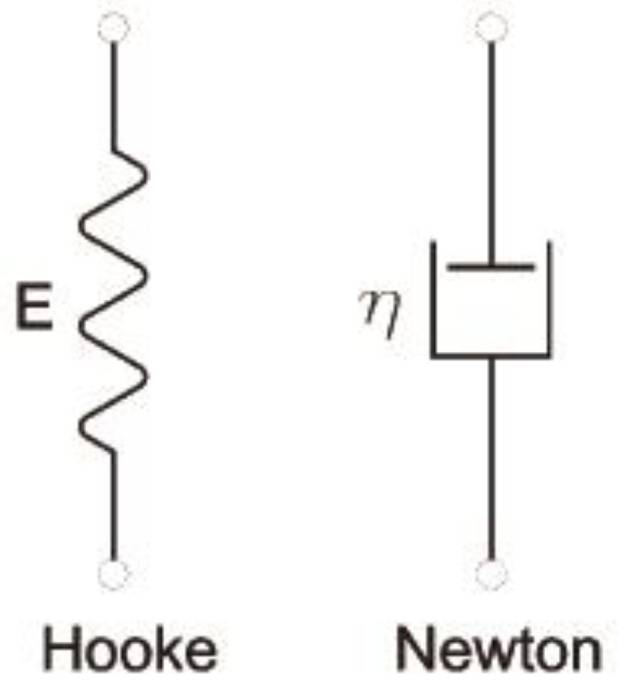
Schemes for Hooke and Newton models.

Figure 2 shows a schematization of both models. Hooke’s elastic element is shown as a spring, while Newton’s viscous element is shown as a damper. In rheology, it is common to work with these representations instead of writing the corresponding equations.

Historically, the elements of Hooke and Newton were combined with the purpose of giving the properties of both ideal materials to more realistic models. Two combinations are possible: in series and in parallel. The Maxwell viscoelastic material model is obtained from the series connection of the two basic elements, and the Voigt model is obtained from the parallel connection. Both models are still quite far from the behavior of real materials.

**Figure 3:**
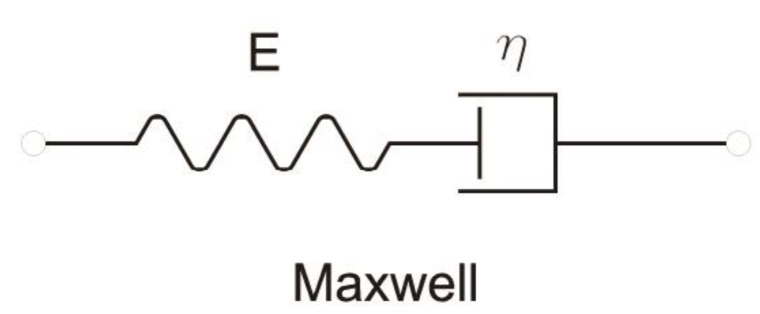
Scheme for Maxwell model.

The case of the Maxwell model (Figure 3) is elucidated by Equation (22).

(22) $$\frac{d\varepsilon }{dt}=\frac{1}{E}\frac{d\sigma }{dt}+\frac{\sigma }{\eta }$$

If *σ* is constant, then $\frac{d\varepsilon }{dt}$ is also constant, which means that if the stress is constant, the strain increases to infinity, which does not correspond to any experimental observation.

**Figure 4:**
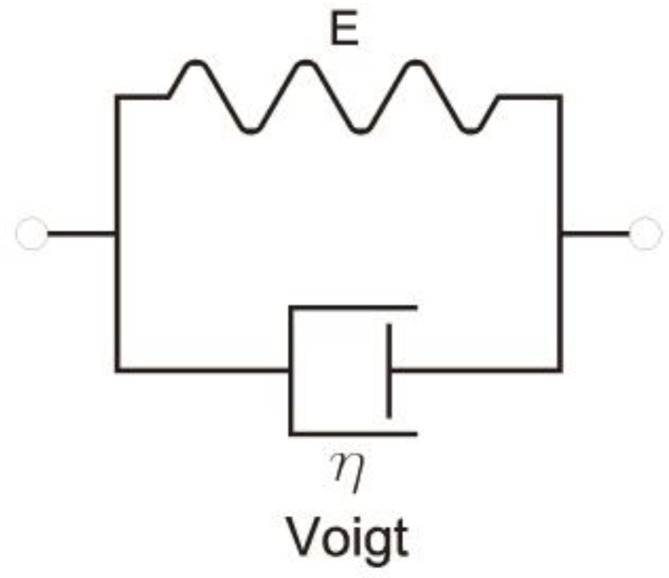
Scheme for Voigt model.

In the case of the Voigt model (Figure 4), *σ* and *ε* are related by the following:

(23) $$\sigma =E\varepsilon +\eta \frac{d\varepsilon }{dt}$$

It follows that if *ε* is constant, then *σ* is constant, so the Voigt model does not reflect the experimentally observed stress relaxation phenomenon.

**Figure 5:**
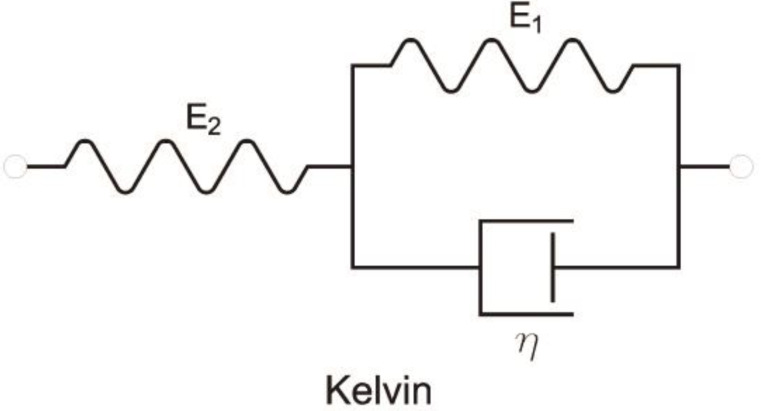
Scheme for Kelvin model.

At the next level of complexity, the disadvantages of the Maxwell and Voigt models have improved. The series coupling of a viscoelastic Voigt element with an elastic Hooke element provides us with the Kelvin model (Figure 5):

(24) $$\frac{d\sigma }{dt}+\frac{E_{1}+E_{2}}{\eta }\sigma =E_{1}\left(\frac{d\varepsilon }{dt}+\frac{E_{2}}{\eta }\varepsilon \right)$$

**Figure 6:**
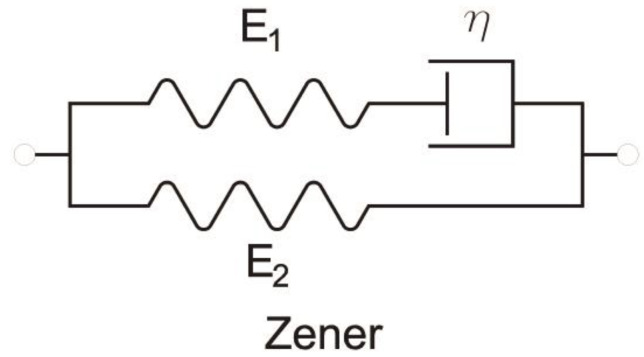
Scheme for Zener model.

By connecting a Maxwell element with a parallel Hooke element, we obtain the Zener model (Figure 6):

(25) $$\frac{d\sigma }{dt}+\frac{E_{2}}{\eta }\sigma =\left(E_{1}+E_{2}\right)\frac{d\varepsilon }{dt}+\frac{E_{1}E_{2}}{\eta }\varepsilon$$

Both the Kelvin model and the Zener model provide a good qualitative description, but are not considered satisfactory from the point of view quantitative [44,45]. For this reason, further rheological models of more complex viscoelastic materials were developed, consisting of the combination of several (sometimes many) Kelvin or Maxwell elements combined with elastic elements of Hooke. These models imply an increasing complexity of the expressions that relate stress and strain, in which linear combinations of the derivatives of any integer order of stress with respect to strain appear. In the most general case, we arrive at a model in the following form:

(26) $$\sum_{k=0}^{n}a_{k}\frac{d^{k}\sigma }{{dt}^{k}}=\sum_{k=0}^{m}b_{k}\frac{d^{k}\varepsilon }{{dt}^{k}}$$

obtaining the best results when *n* = *m* (this property comes from the Kelvin and Zener models, in which *n* = *m* = 1).

Using Equation (26) as the basic law of deformation of viscoelastic materials leads to complicated differential equations and high derivative orders in the formulation and resolution of applied problems, despite the fact that the resulting differential equations are linear (due to the linearity of the basic law of deformation).

However, there is an alternative approach that, while preserving the linearity of the models, provides better application levels.

Indeed, in 1947, Blair [46,47], noting that stress is proportional to the zero-order derivative of deformation in solids and proportional to the first derivative in fluids, proposed intermediate materials for those the stress would be proportional to the intermediate order (non-integer) derivative of the strain, that is,

(27) $$\begin{array}{cc} \sigma \left(t\right)=E\frac{d^{0}\varepsilon \left(t\right)}{{dt}^{0}} & \;\mathrm{Hooke}\\ \sigma \left(t\right)=K_{0}D_{t}^{\alpha }\varepsilon \left(t\right) & \;\mathrm{Blair}\\ \sigma \left(t\right)=\eta \frac{d\varepsilon \left(t\right)}{dt} & \;\mathrm{Newton} \end{array}$$

where *K* and *α* would be constants dependent on the material (0 < α < 1).

At the same time, Gerasimov [48] suggests a similar generalization of the basic law of the deformation, which can be written in terms of the fractional derivative of Rieman–Liouville as

(28) $$\sigma \left(t\right)=\kappa {}_{-\infty }D_{t}^{\alpha }\varepsilon \left(t\right)\;(0<\alpha <1)$$

in which *κ* would play the role of the coefficient *of generalized viscosity*.

A third formulation of a generalization of the basic law of deformation can still be mentioned, due to Slonimsky [49], who in 1961 proposed

(29) $$\varepsilon \left(t\right)=\frac{1}{\kappa }{\;}_{0}D_{t}^{-\alpha }\sigma (t)$$

Under the conditions that, *ε*(0) = 0 and that the functions are equal to zero for *t* < 0, the three Equations (27)–(29) are equivalent.

Besides these approximations, centered on fractional calculus, to linear viscoelasticity, we will also cite two additional closely related approaches.

The above considerations can be considered as a transition from classical linear viscoelasticity based on integer-order models towards fractional-order models from the point of view of the mathematical description of the deformation laws in terms of fractional derivatives. However, fractional-order viscoelasticity models can also be derived from the so-called law stress relaxation potential in real materials, first clearly formulated by Nutting [50] in the following form:

(30) $$\varepsilon =at^{\alpha }\sigma^{b}$$

where *a*, *α*, and *b* are the parameters of the model.

Taking *b* = 1 and setting *C*_0_ = 1/*a*, we see that for a constant strain (*ε* = const.), the stress relaxation is described by the following potential relation:

(31) $$\varepsilon \left(t\right)=\frac{\sigma }{c_{0}}t^{\alpha }$$

As shown by Nonnenmacher [51], from Equation (30) or Equation (31), it follows that the functions *σ*(*t*) and *ε*(*t*) satisfy the following fractional differential equations:

(32) $$D^{\alpha }\sigma \left(t\right)=\frac{\Gamma (1-\alpha )t^{-\alpha }}{\Gamma (1-2\alpha )}\sigma (t)$$

(33) $$D^{\alpha }\varepsilon \left(t\right)=\Gamma (1+\alpha )t^{-\alpha }$$

This highlights the close relationship that exists between the representation of viscoelastic behavior by means of a power law and fractional derivatives.

## 3. Process of Generalization of Concrete Stress–Strain Equations

### 3.1. Introduction

To illustrate the generalization process that has led us to propose the equation we present in this article, we will rely on the series of articles published by Yip et al. between 1995 and 1997 [52,53] in which they explain the proposal by Smith and Young (Equation (1)) by means of the *isoenergy density* theory and Weibull distribution. According to the isoenergy density theory, the concrete could be modeled as a continuum which is elemental in volume, composed of an arbitrarily large set of *isoenergy elements*, each of which is considered linearly elastic, i.e., obeying Hooke’s law, and which may be constituent elements of any of the three phases of concrete, i.e., hardened particles of cement paste, mortar particles (sand-cement), or gravel particles; that is, they can be located at any point in the concrete mixture. The micro-fracturing of the micro-structural material within an isoenergy element would represent the formation of micro-fissures in the concrete.

For the distribution function (developing the usual 1939 Weibull argument), they choose their distribution function with no displacement parameter, obtaining

$$P=1-\exp\left[-{\left(\frac{\varepsilon }{\varepsilon_{0}}\right)}^{m}\right]$$

Stating that the term (*ε*/*ε*_0_)*^m^* must depend on the energy density of the isosurface, *s*, of a micro-fissure that spreads through the heart of the micro-structural material of an isoenergy element and that *s* is a function of the square of the isostrain, *e*, they establish that the value of *m* must be two and write the following:

$$P=1-\exp\left[-{\left(\frac{e}{e_{0}}\right)}^{2}\right]=1-\exp\left[-\frac{s}{s_{0}}\right]$$

Considering that they obtained a straight line by representing *y* = ln ln (1/(1 − *P*)) against *x* = ln (*ε*)^2^ from the experimental results revealed by a compression test of a concrete test specimen, this could be written as follows:

$$P=1-\exp\left[-\frac{\varepsilon }{\varepsilon_{0}}\right]$$

By applying the usual isotropic damage theory argument and remembering that it had been submitted to the perfectly elastic behavior of the isoenergy elements, they arrive at the following equation:

$$F=K\delta \exp\left[-\frac{\varepsilon }{\varepsilon_{0}}\right]\Leftrightarrow \sigma =E\varepsilon \exp\left[-\frac{\varepsilon }{\varepsilon_{0}}\right]$$

where *F* = *σA*, *δ* = *εL*, and *E* = *K*/*AL*, with *A* being the area of the transverse section and *L* being the length of the test specimen, respectively. In order to arrive definitively at Equation (1), it is enough to equate *E* to *f*′*_c_*/*ε*_0_ and set a maximum of *ε* = *ε*_0_ for the function.

To sum up, Yip *rediscovers* the 1956 Smith and Young equation while placing two restrictions: the first consists of making the Weibull distribution exponent *m* equal to the unit (thus restricting the great versatility of this distribution, without a doubt its most remarkable quality) and the second, attributing perfectly elastic behavior to elemental volume particles, which the materials would be composed of at the heart of Continuous Medium Mechanics, thus obeying Hooke’s law.

We, on the other hand, will follow precisely the opposite path to that followed by Yip, i.e., building the equation that models the stress–strain curves from generalizations.

### 3.2. First Generalization: Fractional Hooke’s Law

Any time that Hooke’s law can be expressed as

(34) $$\frac{d\sigma }{d\varepsilon }=E$$

we can (in light of the approaches of the different integral calculus reviewed before) generalize the order of derivation of this differential equation (Equation (34)) and propose a fractional Hooke’s law with non-integer orders of derivation:

(35) Dεα0σ=K

This is obtained by integrating the following:

(36) $$\sigma =\frac{K}{\alpha \Gamma (\alpha )}\varepsilon^{\alpha }$$

Thus, we will use this Equation (36) to represent the behavior of the undamaged material points.

### 3.3. Second Generalization: Exponent m

It appears that the theoretical discourse developed by Yip, based on isoenergy density theory, was designed more to force its results to coincide with the Smith and Young equation than to find an equation to model the largest number of stress–strain curves for the various types of concrete given that, as we have commented, assigning a fixed value to the exponent *m* = 1, means that Weibull distribution loses all its versatility.

Indeed, one of the reasons why Weibull distribution is so widely used is that it is able to model processes in which the failure rate, *h*:

(37) $$h\left(\varepsilon ;\varepsilon_{a},\varepsilon_{0},m\right)=\frac{m}{\varepsilon_{0}}{\left(\frac{\varepsilon -\varepsilon_{a}}{\varepsilon_{0}}\right)}^{m-1}$$

1. Increases with the strain if *m* > 1.

2. Is constant with the strain if *m* = 1 (a hypothesis defended by Yip). 

3. Decreases with the strain if *m* < 1.

For this reason, (in the initial approximation) we will adopt the Weibull distribution to represent the evolution of the damage to the materials as set out by this author keeping its exponent *m* variable for each material and even, as we will see, for each individual specimen.

### 3.4. Third Generalization: Generalized Extreme Value Distribution

We would be going counter to the necessary generalizing spirit of all science if, knowing that a mathematical expression is a particular case of another more general one, we used the former in place of the latter to model a specific physical process using a formula. Although in the initial attempts to propose an equation to model the stress–strain curves for concrete, the use of Weibull distribution as a function of the damage seemed to fit the experimental data quite well, the existence of a generalized distribution to include the majority of known continuous distributions, has led us to adopt it as a distribution function of the damage at our proposal.

As an example of this, we will mention an article written by Basu et al. [54] which appeared in 2009, in which the authors wondered if Weibull distribution is the most appropriate statistical distribution for brittle materials and, to attempt to answer the question, they carried out various tests on different structural ceramic materials and on glass, contrasting the results with four statistical distributions, namely, Weibull distribution, normal distribution, log-normal distribution, gamma distribution, and generalized exponential distribution (due to Gupta and Kundu [55]). As a result of their analysis, they conclude that in some cases, gamma or log-normal distribution, in contrast with Weibull or generalized exponential distribution, seem to describe the strength data measured experimentally more appropriately.

However, as the Fisher–Tippett–Gnedenko theorem allows us to state, the distributions used to compare the data in the article by Basu et al. are nothing more than particular cases (limits) of Generalized Extreme Value Distribution. The form of limit distributions for maxima drawn from samples whose initial distribution is *F*(*x*) is summarized in Table 1:

### 3.5. Formulation of the Stress–Strain Equation: Restricted Form

A damage model based on an internal damage variable represented by a general equation that relates stresses with strains can be expressed by Equation (5), so we propose the following:

1. If we assume that the material is composed of a sufficiently large number of sufficiently small (continuous medium) identical (homogeneous material) *particles*, then *ω* (*ε*) can be considered a random variable with a probability distribution function given by the Generalized Extreme Value distribution function (Equation (13)). Thus, Equation (5) would now be written as follows:

(38) $$\sigma =\Psi \left(\varepsilon \right)\exp\left[-{\left(1+\frac{1}{\xi }\left(\frac{\varepsilon -a_{n}}{b_{n}}\right)\right)}^{-\xi }\right]$$

2. Elsewhere, if the function *Ψ*(*ε*), which represents the behavior of the *particles* of the material in the absence of damage, adopts the fractional form of Hooke’s law in Equation (32), the equation we propose to model the stress–strain curves of concretes tested with a constant rate of increase in stress *up to the point immediately before breaking* takes the following form:

(39) $$\sigma =\frac{K}{\alpha \Gamma (\alpha )}\varepsilon^{\alpha }\exp\left[-{\left(1+\frac{1}{\xi }\left(\frac{\varepsilon -a_{n}}{b_{n}}\right)\right)}^{-\xi }\right]$$

where Γ(*α*) is Euler’s gamma function and *K*, *α*, *a_n_*, *b_n_*, and *ξ* are parameters with no formally attributed physical meaning (for the moment), which are not all necessarily anything other than zero and are to be determined experimentally.

### 3.6. Complete Stress–Strain Curves

In the previous section, we defined Equation (39) as an appropriate equation to model the stress–strain curves for concretes tested with a constant rate of increase in stress *up to the point immediately before it breaks*. So, what happens in the case of a constant rate of increase in strain or beyond the breaking point? Does the equation cease to be valid? What happens in the case of a constant rate of increase in strain beyond the stress peak is (as Blechman suggested) that the test specimen *breaks*, if we understand by breaking a process of instability in the machine–specimen system and a change in the stress–strain properties of the material.

**Figure 7:**
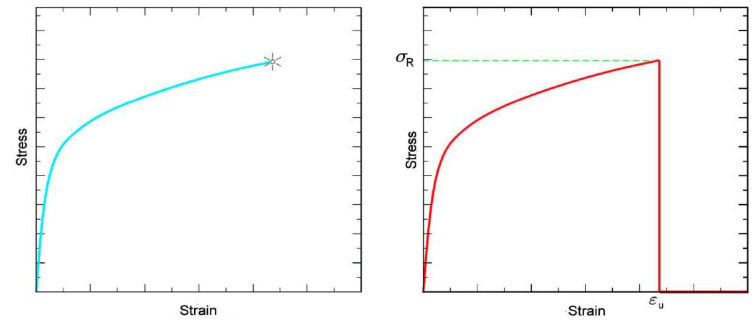
Incomplete (**left**) and complete (**right**) representations of a generic stress–strain curve. (The symbol in the form of an asterisk indicates the breakage of the test specimen).

The stress–strain diagrams in use tend not to represent this fact, but generally only show an asterisk or other symbol at the breaking point, and others simply leave the curve *hanging* (Figure 7 (left)).

However, if we show the complete process, the stress–strain diagram should look similar to that in Figure 7 (right), in which the vertical section indicates the final or breaking strain (*ε_u_*), the breaking stress (*σ_R_*), and the test’s completion, and our function should be able to model this.

**Figure 8:**
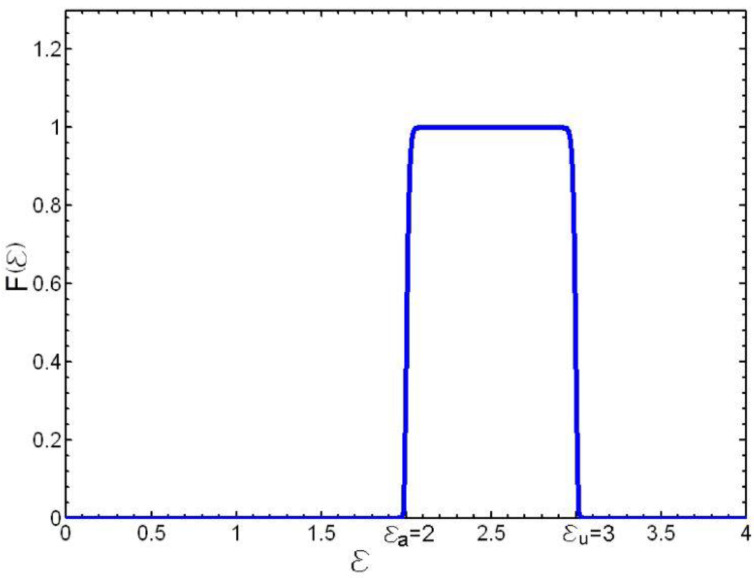
Representation of a potential barrier between *ε_a_* = 2 and *ε_u_* = 3.

To attempt to model this fact that we have observed experimentally (since if we keep the extensometers and the load cell sending data to the capture and storage system, we obtain a representation similar to that in Figure 7 (right)), our thinking will be as follows:

Let us suppose that the test itself is also part of the straining process. Then, the probability can be defined that the test is taking place without interruption. This will equal one while the test specimen has not yet broken and zero following the break, or, seen in another way, we can accept the probability that during the test, no instability has been produced in the system. Then, taking into account the property shown by the *kernel* of the GEVD through which, as |*ξ*| grows and converges on a *potential barrier* (Figure 8) supported by the interval (2 *ε_a_* − *ε_u_* < ε_a_ < ε_u_) with *ε_a_* = *a_u_* − *ξ_u_b_u_* and *ε_u_* = *ξ_u_b_u_*, we can express the probability that the test is being carried out without interruption by means of the distribution function:

(40) $$F(\varepsilon ;a_{u},b_{u},\xi_{u})=\exp\left[-{\left(1+\frac{1}{\xi_{u}}\left(\frac{\varepsilon -a_{u}}{b_{u}}\right)\right)}^{-\xi_{u}}\right]$$

where *ξ_u_* is as big a number as the accuracy and sensitivity of the method used to detect the breaking or instability phenomenon. Figure 8 shows this distribution function for the following parameters: *ε_a_* = 2, *ε_u_* = 3, and *ξ_u_* = −50.

If we are completely sure that the test begins at *ε* = 0, then *ε_a_* = 0, and we can write the following:

(41) $$F(\varepsilon ;a_{u},b_{u},\xi_{u})=\exp\left[-{\left(1+\left(\frac{\varepsilon }{\xi_{u}\varepsilon_{u}}\right)\right)}^{-\xi_{u}}\right]$$

Therefore, a more general equation than Equation (39) can be written to model situations such as that in Figure 7 (right), in the form of

$$\sigma =\frac{K}{\alpha \Gamma (\alpha )}\varepsilon^{\alpha }\exp\left[-{\left(1+\frac{1}{\xi }\left(\frac{\varepsilon -a}{b}\right)\right)}^{-\xi }\right]\exp\left[-{\left(1+\frac{1}{\xi_{u}}\left(\frac{\varepsilon -a_{u}}{b_{u}}\right)\right)}^{-\xi_{u}}\right]$$

in which some of the parameters that we had defined with no physical meaning begin to acquire it. Specifically, if we single out *ε_u_* = *a_u_* − *b_u_ξ_u_* = 0, *a_u_* = *b_u_ξ_u_*, *ε_u_* = *b_u_ξ_u_*, *α_u_* = 0, and *K_u_* = 0, we can write the following:

(42) $$\sigma =\frac{K}{\alpha \Gamma (\alpha )}\varepsilon^{\alpha }\exp\left[-{\left(1+\frac{1}{\xi }\left(\frac{\varepsilon -a}{b}\right)\right)}^{-\xi }\right]\exp\left[-{\left(\frac{\varepsilon }{\varepsilon_{u}}\right)}^{\xi_{u}}\right]$$

which is the equation we propose to model stress–strain curves for concretes from the beginning of the test beyond any point of instability.

### 3.7. General Form of the Equation

As we commented, in the case of the constant rate of increase in strain, following Blechman’s ideas, we find a more complex situation since shortly after the stress peak, instability phenomena are produced in the machine–specimen system due to the macroscopic breaks in the specimens’ experiences. Thus, we have a system in which the material appears to go through two or more phases, and the machine–specimen system appears to experience one or several instability phenomena. Both the various phases the material appears to go through, and the possible instability episodes that can be modeled by means of a more general expression of Equation (39) or Equation (40), namely,

(43) $$\sigma =\sum_{i=1}^{n}\frac{K_{i}\varepsilon^{\alpha_{i}}}{\alpha_{i}\Gamma (\alpha_{i})}\exp\left[-\sum_{j=1}^{m}{\left(1+\frac{1}{\xi_{j}}\left(\frac{\varepsilon -a_{j}}{b_{j}}\right)\right)}^{-\xi_{j}}\right]$$

where *K_i_* ≥ 0, $a_{i}\in R,$ *α_i_* ≥ 0, *b_j_* > 0, and *ξ_j_* < 0 are the parameters (of the materials, the shape and size of the test specimens used to test them and of the temperature, the machines, and test methods) that need to be determined experimentally. Additionally, *n*, *m* ∈R represents the number of *phases* or *subehaviors* which the materials present and the number of *instability processes* or *transitions* which the test specimen goes through during the test, including breaking. The field of application of the Equation (43) can be extended from *before* the test starts until *after* it ends.

In Equation (43) it is possible to perform some algebraic manipulations and, using a more engineering notation, also to express it as follows:

(44) $$\sigma =\sum_{i=1}^{p}C_{i}\varepsilon^{n_{i}}\exp\left[-\sum_{j=1}^{q}{\left(\frac{\varepsilon -\varepsilon_{a_{j}}}{\varepsilon_{0_{j}}}\right)}^{m_{j}}\right]$$

where now *C_i_* = *K_i_*/(*α_i_* Γ(*α_i_*)), *n_i_* = *α_i_*, *ε_aj_* = *a_j_ − ξ_j_b_j_*, *ε*_0*j*_ = *ξ_j_b_j_*, *m_j_* = *−ξ_j_* > 0, *p* = *n* and *q* = *m*.

## 4. Experimental Verification of the Results

We have carried out hundreds of tests to develop this work, for more than ten years, but because in all cases the results have been equally satisfactory, as representative examples, the curves for three generic types of concrete, with low (L1), medium (M1, M2), and high (H1) resistance, have been included. The test specimens were made, cured, and tested at the age of 28 days. The dosages used for the fabrication of the various concretes are shown in Table 2.

### 4.1. Results for the Low-Strength Concrete Test Specimens

**Figure 9:**
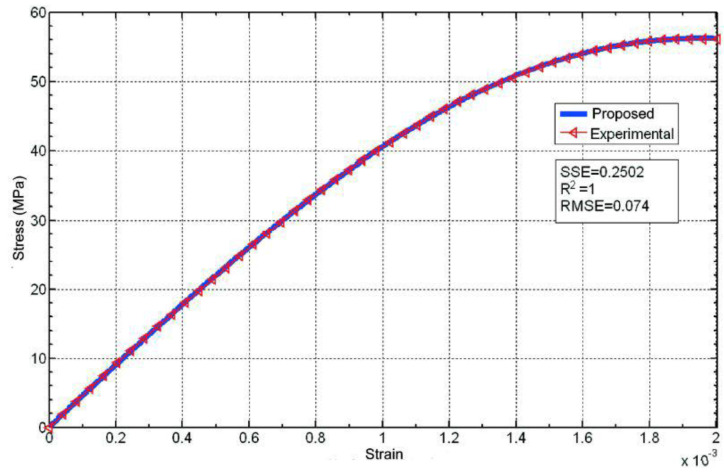
Comparison between the experimental data for test specimen M1 (tested with a constant rate of increase in stress) and the proposed equation.

The fit of the proposed equation to the data experimentally obtained for test specimen M1 (tested with a constant rate of increase in stress equal to 0.6 MPa·s^−1^) shows excellent results (Figure 9) for the following equation:

(45) $$\sigma =K\varepsilon^{n}\exp\left[-{\left(\frac{\varepsilon }{\varepsilon_{0}}\right)}^{m}\right]$$

With *K* = 41,000 MPa, *n* = 0.9885, *ε*_0_ = 0.00282 and *m* = 2.325.

The value of *ε*_0_ should not be confused with the deformation at which fracture occurs or with the deformation at maximum stress. Equation (45) has a maximum when its derivative is equal to zero, that is, when *ε* = *ε*_0_(*n*/*m*)^1/*m*^ (not *ε* = *ε*_0_). According to the results found for the best possible fit of the curve, the maximum would be *ε* = 0.00282 (0.9885/2.325)^(1/2.325)^ = 0.001952 ≅ 0.002, the value of the deformation at which the specimen broke.

The case of a constant rate of increase in strain (2·10^−6^ s^−1^) with which specimen M2 was tested is, as we have shown, somewhat more complex, since a change-of-phase phenomenon is produced after the stress peak, with the material subsequently evolving towards a type of *unstable creep*.

Thus, we have two phases, which is why our equation will be composed of two terms (one for each phase), and the last of them is weighed by an additional exponential, which models the phase change.

The values which best adjust the proposed equations to the experimental data for test specimen M2 are those shown in the following equation:

(46) $$\sigma =C_{1}\varepsilon^{n_{1}}\exp\left[-{\left(\frac{\varepsilon }{\varepsilon_{0_{1}}}\right)}^{m_{1}}\right]+C_{2}\varepsilon^{n_{2}}\exp\left[-{\left(\frac{\varepsilon }{\varepsilon_{0_{2}}}\right)}^{m_{2}}\right]\exp\left[-{\left(\frac{\varepsilon -\varepsilon_{a}}{\varepsilon_{0_{3}}}\right)}^{m_{3}}\right]$$

**Figure 10:**
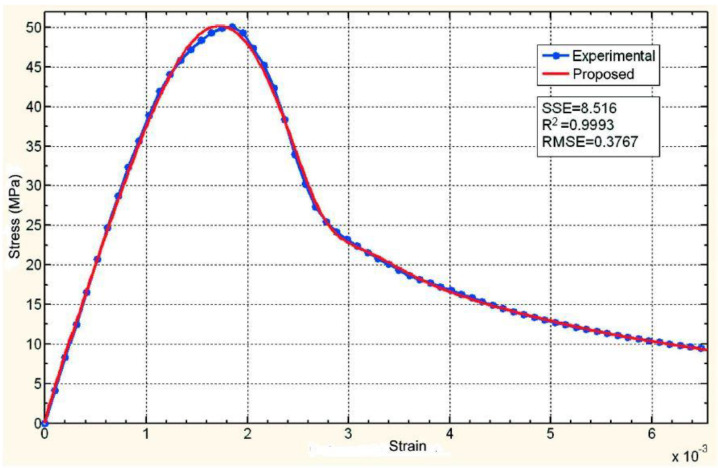
Comparison between the experimental data for test specimen M2 (tested with a constant rate of increase in strain) and the proposed equation.

These are represented in Figure 10 with the following parameters:

Phase I: *C*_1_ = 25,220 Mpa, *n*_1_ = 0.9405, *ε*_01_ = 0.00253, *m*_1_ = 3.708.

Phase II: *C*_2_ = 17,470 Mpa, *n*_2_ = 0.00319, *ε*_02_ = 4.62 × 10^−8^, *m*_2_ = 0.169.

Phase change: *ε*_a_ = 0.3046, *ε*_03_ = 0.3015, *m*_3_ = 962.1.

### 4.2. Results for the Medium-Strength Concrete Test Specimens

The virtual accuracy obtained for all the cases of a constant rate of increase in stress makes it unnecessary to show it for the rest of the types of concrete produced. We will focus, therefore, on the tests with a constant rate of increase in strain.

**Figure 11:**
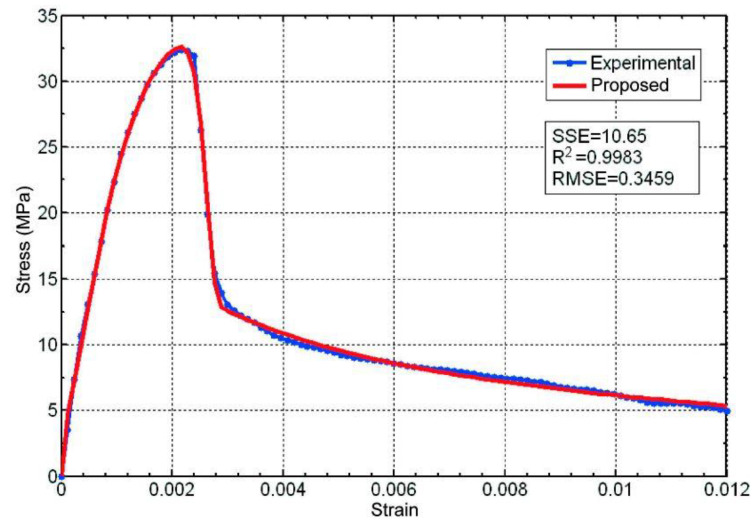
Comparison between the experimental data for test specimen L2 (tested with a constant rate of increase in strain) and the proposed equation.

In the case of test specimen L2, tested with a constant strain increase rate of 6.74 × 10^-6^ s^−1^, a practically vertical section of the curve is observed almost immediately after the stress peak (Figure 11), probably due to the rise in the rate of increase in strain compared to the test with specimen M2, which reveals the existence of an overall instability process which is recovered close to the strain corresponding to 0.003. This fact can be modeled by means of the following equation:

$$\sigma =C_{1}\varepsilon^{n_{1}}\exp\left[-{\left(\frac{\varepsilon }{\varepsilon_{0_{1}}}\right)}^{m_{1}}\right]\exp\left[-{\left(\frac{\varepsilon }{\varepsilon_{0_{2}}}\right)}^{m_{2}}\right]+C_{2}\varepsilon^{n_{2}}\exp\left[-{\left(\frac{\varepsilon }{\varepsilon_{0_{3}}}\right)}^{m_{3}}\right]\exp\left[-{\left(\frac{\varepsilon -\varepsilon_{a}}{\varepsilon_{0_{4}}}\right)}^{m_{4}}\right]$$

with the following parameters:

Phase I: *C*_1_ = 86,260 Mpa, *n*_1_ = 0.978, *ε*_01_ = 0.00541, *m*_1_ = 1.

Instability: *ε*_02_ = 0.000263, *m*_2_ = 19.31.

Phase II: *C*_2_ = 997.6 Mpa, *n*_2_ = 0.185, *ε*_03_ = 10^−5^, *m*_3_ = 0.209.

Phase change: *ε*_a_ = 0.00753, *ε*_04_ = 0.00664, *m*_4_ = 12.28.

### 4.3. Results for the High-Strength Concrete Test Specimens

**Figure 12:**
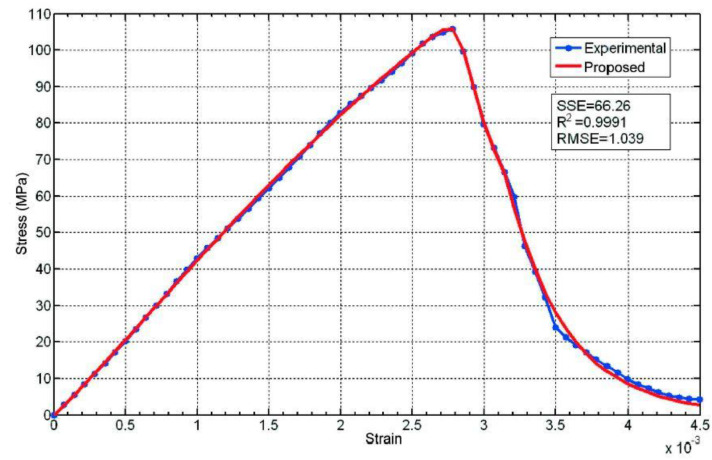
Comparison between the experimental data for test specimen H2 (tested with a constant rate of increase in strain) and the proposed equation.

In Figure 12, it can be observed that high-strength concretes typically present a more *linear* ascending branch, with the descent from the stress immediately after the maximum proving more pronounced.

The final residual stress *tail* (which Blechman attributed to a confinement factor) proves lower than in normal concretes.

The values found for the parameters which best adjust the proposed equation (Equation (44)) to the experimental results for test specimen H2 are the following:

Phase I: *C*_1_ = 33,920 Mpa, *n*_1_ = 0.9695, *ε*_01_ = 0.08598, *m*_1_ = 9.34.

Instability: *ε*_02_ = 9.068·10^−5^, *m*_2_ = 0.6623.

Phase II: *C*_2_ = 2.041 × 10^6^ Mpa, *n*_2_ = 1.036 × 10^−10^, *ε*_03_ = 0.003135, *m*_3_ = 19.64.

Phase change: *ε*_a_ = 0.01165, *ε*_04_ = 0.008511, *m*_4_ = 100.

## 5. Discussion

In the first place, as we have mentioned above, we wish to stress that this work does not advocate a constitutive model.

On the other hand, the proposed equation encompasses the classical laws for materials with infinite ideal resistance as limiting cases, including Hooke’s law, the perfectly plastic behavior law, and the power law (Hollomon’s equation) or its variation (Ludwik’s law).

In particular, when setting *p* = 1, *q* = 1, *C* = e*E*, *α* = 1, and *m* = 0 in Equation (44), we obtain σ = *εE*, which is none other than the famous Hooke’s law. Alternatively, if we fix *p* = 1, *q* = 1, *C* = e*σ_y_*, *α* = 0, and *m* = 0, we attain *σ* = *σ_y_*, corresponding to the perfectly plastic behavior law. Similarly, by setting *p* = 1, *q* = 1, *C* = e*K*, *α* = *n*, and *m* = 0, we arrive at *σ* = *Kε^n^*, known as the power law or Hollomon’s equation.

Finally, when we let *p* = 2, *q* = 1, *C*_1_ = e*σ_y_*, *α*_1_ = 0, *m* = 0, *C*_2_ = e*K*, and *α*_2_ = n, we obtain *σ* = *σ_y_* + *Kε^n^*, which is referred to as Ludwik’s law.

## 6. Conclusions

To conclude, we discuss the principal conclusions that the present research work reveals.

First of all, a fractional version of Hooke’s law is proposed, based on integral calculus, which is why, far from being different, Hooke’s law (Equation (34)) and Bach’s equation are nothing more than particular cases of the fractional differential equation proposed in Equation (44).

Next, taking the assumptions of Continuous Damage Mechanics as the starting point (Equation (5)), an equation is proposed that models the stress–strain evolution of materials that present *simple* behavior, (Equation (36)). Classical laws in which the behavior is always perfect and the strength of the material is infinite, namely, Hooke’s law, that of constant creep stress, and the power law (Bach’s equation), are limited cases of this equation.

For those cases in which the material appears to go through two or more phases and the machine–specimen system appears to experience one or several instability phenomena, the stress–strain curves can be modeled using a more general expression (Equation (44)):

(47) $$\sigma =\sum_{i=1}^{p}{\;C}_{i}\varepsilon^{n_{i}}exp[-\sum_{j=1}^{q}{\;(\frac{\varepsilon -\varepsilon_{aj}}{\varepsilon_{0j}})}^{m_{j}}]$$

where *C_i_*, *n_i_*, *ε_aj_*, *ε_0j_*, and *m_j_* are the parameters (of the materials, the shape and size of the test specimens used to test them and of the temperature, machines, and test methods) to be determined experimentally, *p* and *q* are natural numbers and represents the number of *phases* or *sub-behaviors* the material presents and the number of *instability processes* or *transitions* the machine–specimen system goes through during the test, including breaking.

**Figure 13:**
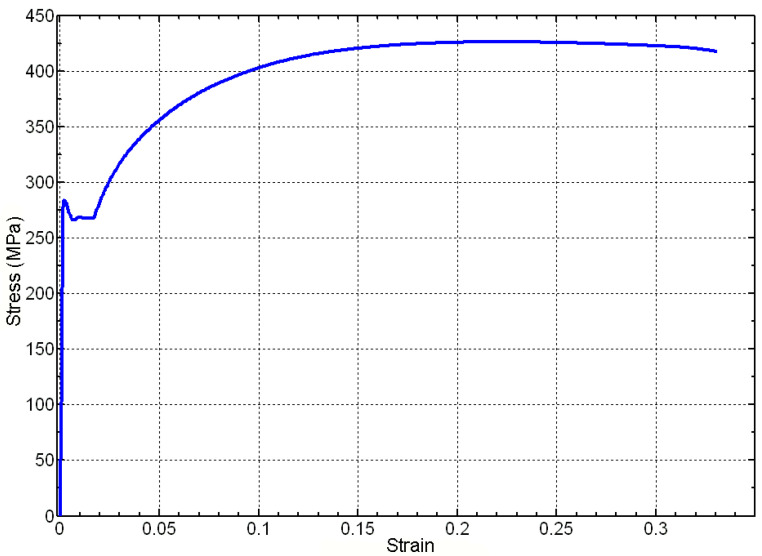
Typical curve of the stress–strain behavior of structural steel (Source by the authors) [56,57].

Trying to clarify the physical meaning of the proposed equation, let us take the well-known example of the stress–strain curve of common construction steel. To put it briefly, carbon steel is a binary alloy of Fe-C that typically contains less than 1% carbon, with carbon content ranging between 0.008% and 2.11%. At room temperature, it exhibits a stable structure consisting of grains of *ferrite* or Fe-α phase, with colonies of iron carbide or *cementite* dispersed throughout. Cementite is an interstitial solute in iron and forms solid solutions with ferrite, while Fe-α has a Centered Cubic Inside (CCI) structure with very little Fe_3_C at the interstices of its grains. Carbon significantly affects the mechanical properties of steel, with the Fe-α phase being relatively soft and ductile, and cementite being hard and brittle. The characteristic stress–strain diagram of these steels, featuring upper and lower yield points, a plastic plateau (with or without the Portevin–Le Chatelier effect), and later hardening by strain, can be attributed to the interplay between these two behaviors, as depicted in Figure 13.

It appears as though the behavior of the system can be modeled in a way that does not require modifying the proposed equation and without the need for separate expressions for elastic and plastic strain, as is typically carried out in classical theory. The particles of the weaker phase (cementite) appear to break under stress, following Weibull’s weakest link theory, which allows for a sharp increase in stress on the stronger phase (ferrite) particles. This causes instability in the machine-probe system, resulting in yield points and a plastic plateau. Eventually, the stresses rearrange themselves, and the system becomes stable again, with stress increasing once more, but now exhibiting plastic behavior that is more typical of the ferrite phase. In light of this, it is worth asking whether it is possible to expand the proposed equation in order to model this behavior in a way that does not require the use of separate expressions for elastic and plastic strains. The answer to this question is yes, as expected.

If we consider steel as a material composed of two phases, with behavior laws for their particles, $C_{1}\varepsilon^{n_{1}}$ and $C_{2}\varepsilon^{n_{2}}$, and damage probability distribution functions, $\omega_{1}(\varepsilon ;\varepsilon_{a_{1}},\varepsilon_{0_{1}},m_{1})$ and $\omega_{2}(\varepsilon ;\varepsilon_{a_{2}},\varepsilon_{0_{2}},m_{2})$, we can propose a stress–strain equation as follows:

$$\sigma =C_{1}\varepsilon^{n_{1}}\exp+\varepsilon^{n_{2}}\exp\left[-{\left(\frac{\varepsilon -\varepsilon_{a_{2}}}{\varepsilon_{0_{2}}}\right)}^{m_{2}}\right]$$

**Figure 14:**
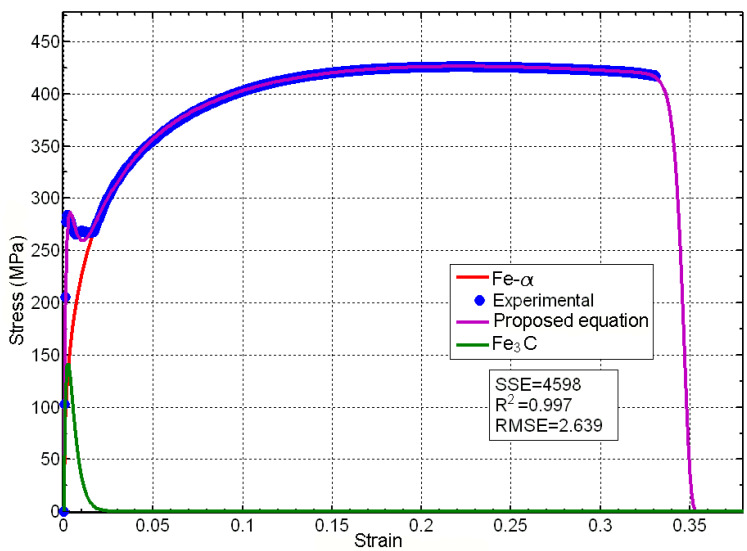
Comparison among the experimental data of Kato et al. and a first approach to the problem by the proposed equation. (Source by the authors) [56,57].

In order to contrast the goodness of the equation, we compared it with the experimental data taken from the article of Kato et al. [58], obtaining the provisional results shown in Figure 14, where the behavior assigned by the equation for phase Fe-_ is represented in red, the behavior for Fe_3_C is represented in green, and the total curve that resulted from applying the proposed equation is represented in magenta. These were obtained for the following values:

*K*_1_ = 184,800 Mpa, _*n*_1_ = 1, *ε_a_*_1_ = 0, *ε*_01_ = 0.002, *m*_1_ = 0.866 and *K*_2_ = 2330 Mpa, _*n*_2_ = 0.4662, *ε_a_*_2_ = 0, *ε*_02_ = 0.1696, *m*_2_ = 0.416.

Finally, we checked that Equation (44) models not only the stress–strain curves which we designate as *simple* but also the curves for the concrete tested with a constant rate of increase in stress, and those that present multiphase behavior, as well as those for concretes tested with a constant rate of increase in strain over a wide range of characteristic strengths, and all with an excellent level of accuracy.

Equation (44), to the best of our knowledge, is the equation that best fits the experimental data from uniaxial compression stress (mean)-strain (mean) tests of any type of concrete tested under any rate of increase in stress or strain and it is continuous and derivable at all its points, so valuable information can be extracted from it, such as the true value of the modulus of elasticity at any point by means of a simple derivation process. On the other hand, it allows for evaluating the failure rate to implement reliability coefficients, calculate strain energies (through integration), and calculate any other information that can be extracted by providing a continuous and differentiable curve at all points that fits as much as we want to the experimental results. We have not found this last extreme in the bibliography, to the best of our knowledge.

## Figures and Tables

**Table 1:** Form of the limit distributions for maxima drawn from samples whose initial distribution is *F*(*x*).

Initial Distribution *F*(*x*)	Limit Distribution for the Maxima *G*(*x*)
Exponential	Type I GEVD (Gumbel)
Gamma	Type I GEVD (Gumbel)
Normal	Type I GEVD (Gumbel)
Log-normal	Type I GEVD (Gumbel)
Pareto	Type II GEVD (Fréchet)
Cauchy	Type II GEVD (Fréchet)
Burr	Type II GEVD (Fréchet)
Log-gamma	Type II GEVD (Fréchet)
Uniform	Type III GEVD (Weibull)
Beta	Type III GEVD (Weibull)

**Table 2:** Design of the mixtures of the concretes tested.

Test Specimen	CEM (kg/m^3^)	W/C	CA (kg/m^3^)	FA (kg/m^3^)	SF (kg/m^3^)	WR(kg/m^3^)	*f*′*c* (MPa)
L2	250	0.65	975.41	1056.46	-	-	32.35
M1	375	0.44	925.76	995.71	4.69	-	56.18
M2	375	0.44	925.76	995.71	4.69	-	50.06
H2	500	0.23	907.80	982.26	25.00	12.5	105.78

CEM—Cement, W/C—Water/cement ratio, CA—Coarse aggregate, FA—Fine aggregate, SF—Silica fume, WR—Water reducer/super plasticizer, *f*′*c*—Maximum compressive strength.

## Data Availability

No new data were created or analyzed in this study. Data sharing is not applicable to this article.
